# Balancing collective responsibility, individual opportunities and risks: a qualitative study on how police officers reason around volunteering in an HIV vaccine trial in Dar es Salaam, Tanzania

**DOI:** 10.1186/1471-2458-10-292

**Published:** 2010-05-28

**Authors:** Edith AM Tarimo, Anna Thorson, Thecla W Kohi, Joachim Mwami, Muhammad Bakari, Eric Sandström, Asli Kulane

**Affiliations:** 1 Division of Global Health, Department of Public Health Sciences, Karolinska Institutet, Stockholm, Sweden; 2 Department of Nursing Management, Muhimbili University of Health and Allied Sciences, Dar es Salaam, Tanzania; 3 Department of Sociology and Anthropology, University of Dar es Salaam, Dar es Salaam, Tanzania; 4 Department of Internal Medicine, Muhimbili University of Health and Allied Sciences, Dar es Salaam, Tanzania; 5 Venhälsan, Karolinska Institutet, Södersjukhuset, Stockholm, Sweden

## Abstract

**Background:**

Results from HIV vaccine trials on potential volunteers will contribute to global efforts to develop an HIV vaccine. The purpose of this study among police officers in Dar es Salaam, Tanzania, was to explore the underlying reasons that induce people to enrol in an HIV vaccine trial.

**Methods:**

We conducted discussions with eight focus groups, containing a total of 66 police officers. The information collected was analyzed using interpretive description.

**Results:**

The results showed that participants were motivated to participate in the trial by altruism, and that the participants experienced some concerns about their participation. They stated that altruism in the fight against HIV infection was the main reason for enrolling in the trial. However, young participants were seriously concerned about a possible loss of close relationships if they enrolled in the HIV vaccine trial. Both men and women feared the effect of the trial on their reproductive biology, and they feared interference with pregnancy norms. They were unsure about risks such as the risks of acquiring HIV infection and of suffering physical harm, and they were unsure of the intentions of the researchers conducting the trial. Further, enrolling in the trial required medical examination, and this led some participants to fear that unknown diseases would be revealed. Other participants, however, saw an opportunity to obtain free health services.

**Conclusions:**

We have shown that specific fears are important concerns when recruiting volunteers to an HIV vaccine trial. More knowledge is needed to determine participants' views and to ensure that they understand the conduct of the trial and the reasons it is being carried out.

## Background

The search for an effective HIV vaccine through trials is being actively pursued throughout the world. An extensive body of literature is available that provides knowledge about the factors that influence willingness to participate in HIV vaccine trials. Most of these studies are from high and middle-income countries. Some of these studies have focused on populations at high risk [1-4], while others have devoted attention to other groups in the general population [5-8]. Few studies have looked into willingness in low-income countries in Africa[9-13].

It is important to examine people's reasons for participating in clinical trials in different contexts, given that the reasons not to enrol in HIV vaccine trials may differ. Studies from high-income countries have identified a number of such reasons, including a fear of vaccine-induced HIV infection [4,14], a fear of negative side effects of the vaccine [7,15,16], and worries about what others will think or say about the participants [1]. In South Africa, the major reasons for not participating in HIV vaccine trials are fears that the vaccine may not be safe [5] and a lack of information about vaccines [6]. In addition, community members in Africa may view vaccine research in various ways: they may suspect, for example, that vaccines are a means of eliminating black people by infecting them with AIDS [6]. Others may agree to take part in a clinical trial while holding an opinion that is not supported by the research protocol. In Gambia, parents experienced a clinical trial as a route to better and cheaper medical treatment for their children [17]. Some studies have noted that there is an increasing demand for basic HIV vaccine education to address certain vaccine trial concepts [10,11,18,19].

Tanzania has an HIV prevalence of 6.2% in the population segment of 15-49 years old [20], and is one of the low-income countries in Africa that is conducting HIV vaccine trials [21]. A large HIV incidence study was conducted among police officers, leading to a joint Phase I and Phase II HIV vaccine trial. In a substudy of 329 police officers, 127 (39%) were not willing to participate in a Phase I and Phase II HIV vaccine trial [22]. This study provided useful information, but did not examine in depth the reasons that people refrained from enrolling in the trial. Thus, the aim of the work presented here was to examine how police officers reason around their decision whether to volunteer or not in the HIV vaccine trial in Tanzania. This study has used an interpretive description (ID) approach [23]. ID can provide contextual understanding of the factors that influence the decision of a police officer whether or not to take part in an HIV vaccine trial.

## Methods

### Study area and population

The study was conducted in eight out of 32 police stations according to the availability of study participants in Dar es Salaam, Tanzania. Details of the study population have been presented elsewhere [22,24]. This study was a substudy of a larger Tanzania and Sweden (TANSWED) HIV Program in Tanzania funded by Sida/SAREC. This program includes studies of HIV incidence, studies of laboratory reference values, and the analysis of willingness to participate in HIV vaccine trials described here. An extension of the program includes Phase I and II HIV vaccine trials, as mentioned earlier. Police officers were chosen for the study because they are well educated (most of them have taken four years of secondary education), come from an established organisation and are easy to access. Previous studies had shown that they can make independent informed decisions whether to take part in HIV incidence studies and HIV vaccine trials that have gained the support of higher police authorities [22,24].

### Recruitment

Study participants were recruited based on information from a previous study among police officers [22]. Of the 594 potential participants, 408 (68.6%) signed up for HIV and AIDS education followed by an HIV vaccine trial workshop. The field team (doctors, counsellors and nurses) provided information about HIV and AIDS during the workshop, described the HIV vaccine trial, and described the potential benefits and risks of taking part. Such benefits were: complete medical check up, free medical services and referral to credited health centres if a participant fell sick during the trial, follow-up visits, and regular HIV testing. Risks included possible reactions such as mild pain or tenderness, local redness, and various systemic symptoms such as malaise, headache, mild fever, and nausea [25]. The team clarified the inclusion criteria, which included an age between 18 and 40 years and an undertaking to use effective contraception throughout the trial period.

### Study design and sampling

This was an explorative study conducted among a subgroup of the 408 police officers, a month after the workshop. The aim was to recruit groups of diverse membership with respect to age, sex, rank, location of the police station, marital status and whether the participant had a caregiving role for a relative with AIDS. We intended that such groups would create shared perspectives. The first author (EAMT) characterized the 408 participants according to the predetermined criteria, except for whether the participant was a caregiver (those who cared for a relative with AIDS in their families, see below). EAMT, the fourth author (JM) and two field assistants, a doctor and a nurse from the police health department, visited the selected stations to explain the objectives of the study and to ask for appointments. Participants at these stations who met the inclusion criteria for the substudy were recruited. The two field assistants helped to recruit caregivers after the latter had been fully informed about the focus, objectives, methodological approaches and sensitivity of the study. The field assistants occasionally used the snowball sampling strategy [26] to come into contact with those who care for people with AIDS in their families. The aim of recruiting persons who cared for an AIDS patient was to obtain their views about the topic. A total of 66 participants was recruited, and formed a purposive sample [27] for this substudy (Table 1).

**Table 1:** Participants in the focus group discussions (FGDs)

Group	Inclusion	Participants
1	Unmarried policewomen 30 years old or younger (Young policewomen)	7
2	Policewomen older than 30 years, of low rank (3 of these were married) (Older policewomen)	4
3	Low ranking policewomen of any age, 10 unmarried and 2 married (Low ranking policewomen)	12
4	Unmarried policemen 30 years old or younger (Young policemen)	12
5	High ranking policemen, all above 30 years old and married (High ranking policemen)	7
6	Low ranking policemen, all above 30 years old, half of them were married (Low ranking policemen)	6
7	Married and older policemen, mixed ranks (Married policemen)	12
8	Police officers who have close contact with a relative with AIDS, 3 unmarried men and 3 married women (Caregivers)	6

Total		66

### Data collection procedures

We collected data between November 2006 and January 2007 using focus group discussions (FGDs). The memberships of the FGD groups had been predetermined, but the information collected was based on the principle of 'theoretical saturation' [28]. We therefore stopped enrolling new groups when the information became repetitive and it became clear that there was little to gain by including more participants. The FGD guide was initially prepared in English and then translated into Kiswahili before use in the field. The guide comprised of general socio-demographic background information and posed two main research questions: (1) Can you tell me your views about the problem of HIV and AIDS in the police force? (2) What are the cultural norms, views and opinions among police officers that may influence willingness to volunteer for an HIV vaccine trial? The guide was reformulated after the first two FGDs, to include emerging perspectives such as the research questions: Who should be told and why, if one decides to take part in the trial?, and What opinions do you expect to see in other people? We focus on the cultural norms, views and opinions among police officers that may influence willingness to take part in an HIV vaccine trial in the present report. Findings from the first question will be presented elsewhere. All FGDs were conducted at a location in the police stations selected by the participants, and the number of participants varied between groups (Table 1). The FGDs lasted between 25 and 71 minutes. The first author (EAMT) moderated the FGDs and acted as a notetaker interchangeably with JM. We have therefore applied triangulation [27] of researchers (EAMT and JM) in data collection in order to ensure consistency.

### Ethical considerations

Participants were recruited after ethical approval had been granted by the Muhimbili University of Health and Allied Sciences' (MUHAS) Institutional Review Board, and with the permission of the police authority. The first author and moderator of FGDs reviewed the informed consent form with potential participants and described the principles of voluntary participation, anonymity and the right to withdraw without any consequences for the individual. The need to tape-record the discussion was also explained. We employed multiple steps in obtaining informed consent before the discussions took place (written consent and oral consent, preceded by review of the written consent with potential participants) to ensure understanding and voluntary participation. All potential participants gave their informed consent.

### Data analysis

Analysis followed the interpretive description approach informed by principles of thematic content analysis [23] through an inductive design. The audio-taped FGDs were transcribed verbatim and translated from Kiswahili to English. EAMT listened to the tapes, and compared the Kiswahili version with the English version to ensure accuracy. She repeatedly read all the transcripts to understand the dataset, identify ideas, patterns and codes. The information on the transcripts was inductively coded separately by EAMT and the last author [AK], and then discussed, in order to increase the validity and ensure the quality of the analysis. There were no major differences. This was followed by reading and comparing the codes and developing categories and themes. The team then discussed the preliminary findings with doctors, nurses, counsellors and the two field assistants. The discussion brought more insight into the interpretation. We have therefore applied triangulation of researchers (EAMT and AK) in data analysis in order to ensure consistency. The interpretive process allowed the categories to be organized into two subthemes and one main theme. The categories, subthemes and the main theme were compared with the FGD transcripts to ensure that interpretive analysis reflects the content of the raw data. Finally, all authors read the analysis and agreed on the categories that had emerged. Quotations are given below to reflect the voices of the participants.

## Results

### Participants

Of the 66 police officers, 35 were above 30 years old, while the youngest participant was 19 years old. Most participants were males and single (Table 1). The majority had at least four years of secondary education.

### Theme and subthemes

One main theme emerged that described the reason that could contribute to police officers' decision to take part in the HIV vaccine trial: 'Balancing collective responsibility and individual concerns'. The theme includes subthemes and categories (Table 2). The first subtheme, 'collectivism in the HIV vaccine trial participation', highlights individual motivations, altruism, concerns for significant others (parents, sexual partners, relatives, colleagues and friends), reproduction and pregnancy norms, and a feeling of responsibility for others in the efforts to save lives from HIV infection. The second subtheme, 'uncertainty of risks and opportunities', draws attention to the adverse effects that may arise from participation in the trial and the contrast between the perceived benefits and the assurance of the researchers on the one hand, and mistrust on the other hand. The theme and its subthemes are presented with some quotations to illustrate these interactions.

**Table 2:** Theme, subthemes and categories

Main theme	Balancing collective responsibility and individual concerns	
Subthemes	Collectivism in HIV vaccine trial participation	Uncertainty of risks and opportunities

Categories	Individual motivations	Risks for HIV infection, myths and vaccine side effects
	
	Altruism	Time of knowing health status
	
	Concerns for significant others (parents, sexual partners, relatives, colleagues and friends)	Medical check up: fear versus opportunities
	
	Reproduction and pregnancy norms	Researchers' assurance and mistrust

	Responsibility for others	

### Collectivism in the HIV vaccine trial participation

#### Individual motivations

Participants articulated several motives for participating in the trial. These included: personal pride, and confidence in a decision to participate in the trial. Some quotations illustrate these motivations:

*I will personally see myself as a hero (Young policeman 2, Group 4)*.

*I will be confident and able to say 'I was among the participants' [in the HIV vaccine trial] (Young policeman 3, Group 4)*.

Participants expected to gain recognition that they felt they deserved after taking part in an exercise that aimed at saving the nation. They wanted to show off that they had helped to make the HIV vaccine trial successful:

*There is a saying: 'He who plays in his or her home ground gets a reward...' I will be proud of myself, like those who fought for independence, that I was among those who fought for the vaccine (Low ranking policewoman 1, Group 3)*.

#### Altruism

In addition, they referred to their obligation as police officers, that the role of protecting civilians could motivate them to take part in the trial. So, they included, fulfilling moral principles and self-sacrifice to save lives of millions of people who are dying of HIV infection:

*I think this [taking part in the trial] is part of motivation in my duty because if I get vaccinated and make it successful, I will save the civilians whom I protect. And to work as a police officer, there must be people to protect. No police force without people. I think this is one of the moral principles that I should do (Low ranking policewoman 10, Group 3)*.

Participants from other groups emphasised other positive results that would be experienced from the trial participation:

*When the results are good and successful, as a participant I will feel like the one who makes success in the trial. I will be proud for making vaccine successful. I will be proud in the community for making the trial successful (Low ranking policeman 2, Group 6)*.

The young policewomen based their altruistic reasons for taking part in the trial on examples from religion. They felt very responsible, and saw their participation as a sacrifice for others:

*I can imagine the vaccine like what Christians say: Jesus was supposed to be crucified on the cross to save lives of others, and that person [a volunteer] will be the victory of saving the nation and the world (Young policewoman 5, Group 1)*.

In contrast, an older policewoman argued that religious issues and participation in the HIV vaccine trial are conflicting issues. She saw this conflict from the fact that the trial rules required participants to postpone giving birth to children, which is contrary to their religious beliefs. She explained:

*And we know that with religion, God said 'Give birth to children, to multiply'. So when it comes to religion, you [researchers] differ ... It is important (Older policewoman 3, Group 2)*.

Overall, participants expressed their reason for taking part is to help to make the trial successful.

#### Concern for significant others

As a counterweight to altruism, the fear of possible loss of close relationships was mentioned as a reason for not volunteering in the trial. The participants signalled that they were confused by the reactions from their friends about their decision to volunteer in the trial:

*I volunteer for the vaccine. Where I go friends will start telling me 'You were healthy and you have been given AIDS'. If they say so, I will be affected psychologically (Young policeman 4, Group 4)*.

The low ranking policewomen felt that colleagues warned them against participation when they shared their intent to take part in the trial. The colleagues warned that they could get AIDS from the trial and would thus be abandoned. Young policewomen also described meeting similar reactions, and subsequently deciding not to participate to avoid being abandoned:

*Every time I told my colleague policewomen about this [the HIV vaccine trial] they challenged me.... 'Go first and get it [the vaccine] because you are HIV positive.'... So I decided to quit... (Young policewoman 2, Group 1)*.

Other participants in the group agreed and supported this discussion through gestures and body language.

Those living with their parents or spouses, and those who had a fiancée said that they feared that participating in the trial might disrupt existing close relationships. Women feared that their intent to take part would signal to their male partners that they have an HIV infection. They said that these opinions could create conflicts with their partners. Young men, in particular, expressed concern about their intimate relationships:

*The fiancée won't trust you from the moment you plan to get the vaccine. She will think that by taking part you are infected straight away ... She will believe that you have been given the virus and so she will also be infected (Young policeman 2, Group 4)*.

Another participant argued that problems with a fiancée arise only if she does not understand about the HIV vaccine trial. He added:

*I think there will be a problem with the fiancée because I have one. Perhaps I will explain to her that I have joined the HIV vaccine trial. If I will not explain properly to make her understand, everything will be interrupted... that will be the end of the relationship... (Young policeman 9, Group 4)*.

Others in the group laughed and nodded as a sign of an experience to identify with.

Existing social relationships and dependencies (parents expecting the young people to support them in their old age) were described as barriers to participating in the trial. The participants said that parents have a strong influence on the lives of young people. Young people saw themselves as an asset for the future with responsibilities towards their parents. These young people speculated that parents would expect them not to take part in the trial since they depend on each other. The parents were said to worry that the vaccine could cause side effects:

*Some of us are taken care of by our parents. Now what would your father say if you get problems? They [parents] will query: 'Don't you know that we depend on you?' (Young policeman 6, Group 4)*.

The caregivers also experienced resistance from relatives against their taking part in the vaccine trial. Their relatives in some cases stated that taking part in the trial would be like killing oneself. Participants suspected that these relatives would warn them against enrolling in the trial because of unknown side effects. The participants became more worried when knowledgeable people cautioned them. One participant shared:

*Personally, I came across such a situation two months after the seminar. I took my documents home [the HIV vaccine trial education materials]. When my relatives read those documents, they were the first ones to warn me: 'Leave it [the trial], and leave it completely'. Many of them, including a doctor from a cancer institute... Now even a person with education became the first one to oppose me. What about those with no education? (Caregiver 1, Group 8)*.

Nevertheless, members of the caregiver group said that some of their relatives would support participation in the trial if they understood the importance of the vaccines. They related this positive assumption based on the experiences of people who take their children to receive available vaccines to prevent various diseases. Likewise, it is possible that they could motivate others to participate in an HIV vaccine trial by referring to a case of HIV infection.

#### Reproduction and pregnancy norms

Participants voiced concern over restrictions imposed while participating in the trial that would threaten the desire to become pregnant. More often they were concerned about the views of others on the effects of taking part in the HIV vaccine trial on their reproductive ability. Both men and women in the study expressed a fear of postponing pregnancy and described how they had been subject to negative views from others. Young unmarried men without children expressed a loss of confidence in the vaccine trial after recalling parents' warnings:

*A parent can tell you that: 'If you are vaccinated and get married you will not get children'. He or she thinks if you get the vaccine you will not get a child... (Young policeman 1, Group 4)*.

Another participant emphasised:

*If you get vaccinated you will not get children when you get married ... you will not get a child... (Young policeman 5, Group 4)*.

Older policewomen also expressed concern over the vaccine rules on pregnancy norms and the possible reactions of sexual partners. These women feared that postponing pregnancy because of enrolment in the vaccine trial could be a major concern to their husbands and boyfriends:

*The problem is with the family [husband], that is when it becomes difficult. I get the vaccine and my husband wants a child then ... I think problems will start there (Older policewoman 1, Group 2)*.

Another group member stated:

*There is a problem with girlfriend and boyfriend. For example, with the boyfriend he could stay with a woman for a long time, outside marriage lock and he wants to have a child with her ... if she postpones, of course the friendship will end (Older policewoman 4, Group 2)*.

They also raised personal doubts about their reproductive capacity:

*Is there any possibility for me to get a child? Won't they [the researchers] just destroy my gametes! (Older policewoman 3, Group 2)*.

Young women reasoned around the possibility of becoming infertile after taking part in the trial:

*There are some women without a child and other people tell them that following vaccination they will never conceive! (Young policewoman 7, Group 1)*.

In addition, the low ranking policemen expressed concern over possible side effects of the vaccine on reproductive health, including foetal abnormalities and impotence in men. They suspected that children might be born with abnormalities because of drugs such as oral contraceptives. They reasoned that similar effects could arise from the HIV vaccine. They also suspected the vaccine could make men impotent:

*People know exactly that children are being born with abnormalities because of drugs [contraceptives]. And now an HIV vaccine has arrived! (Low ranking policeman 3, Group 6)*.

Another policeman added:

*People are thinking a lot about this vaccine, some say why should we be tested with this vaccine? Why not the whole country? Another one says this will finish us, another one that they [the researchers] want to make us impotent ... (Low ranking policeman 5, Group 6)*.

Thus, fear among participants was connected with reproduction and pregnancy issues.

#### Responsibility for others

Another factor for which participants felt responsible was their families, particularly their children. They worried that an experimental vaccine may have a negative outcome, in the extreme case death, if the trial failed. Older married men felt that they were consciously accepting the possibility of death by taking part in the trial, and that this may, in turn, affect the children's future. Although they accepted that death is natural, they said they would prefer a natural death:

*Psychologically it will be bad. We know dying is unavoidable but you can never go and stand on the road and let the car knock you down and you die! Nobody wants to die. All of us here [he pointed to others in the group], we want to stay with our children until they see the last tooth getting out... (Married policeman 1, Group 7)*.

Lack of insurance was described as aggravating the consequences of death that may result from taking part in the trial. They requested that life insurance for their families be guaranteed following their decision to take part in the trial:

*Will I be insured if I die after introducing the virus? What will I leave my children with? We have African families ... One thinks that if I die after being vaccinated; won't I leave them [the family] in difficulties? I better not go for vaccine to be implanted [with HIV in my blood]... (Low ranking policewoman 1, Group 3)*.

Others in the group confirmed the possibility that people would not dare to take part in the trial because of uncertainties about their children's future in the event of a vaccine-related death.

### Uncertainty of risks and opportunities

#### Risks for HIV infection, myths about the vaccine's side effects

Participants expressed a fear of negative outcomes from an experimental vaccine. They believed that the outcome of the vaccination was uncertain. They expressed worries that the trial would affect them in diverse ways. They believed that the vaccine consisted of two shots, the first one containing the HIV virus and the second [boost] to erase the HIV infection. The males shared:

*I think one will not take part in the trial because in the trial viruses must be introduced. If it is for HIV, you are given that vaccine... (Young policeman 12, Group 4)*.

Another participant added:

*How can you protect something [a healthy person] without something [the existence of HIV]? I use a gate because I know thieves might come inside to steal. So how can you put a vaccine if you don't have HIV? The main belief is that you cannot protect something that does not exist (Young policeman 5, Group 4)*.

The participants believed that HIV could be transmitted through either the vaccine or unsafe sex. They reasoned that getting HIV through the vaccine involves pain; while with unsafe sex it involves pleasure. Married policemen explained why a fear of HIV transmission by unsafe sex should differ from that of the vaccine. One participant commented:

*There [in sexual intercourse] you enjoy. That is what it means. It is not like when you are being vaccinated. There [sex] you know you are straightforward enjoying. So it is right if I get infected (Married policeman 7, Group 7)*.

Another participant added another aspect of infection through sex:

*You know with sex, it is imaginary. One goes thinking that he is safe and may be also my partner is safe. You see when doing sex, one does not know... That is the way it is, but sex and vaccine differ a little bit (Married policeman 2, Group 7)*.

Another fear arose from the belief that participating in the trial would introduce the HIV to participants, who would in turn infect their spouses.

In other groups, participants had different views on how vaccines work. They perceived that an HIV vaccine might contain the live virus, which could cause HIV infection, and wondered about how the same vaccine could provide protection. They shared their perception in the following way:

*Most people say you will be given the HIV vaccine first, and then you get the medicines. Others said after being vaccinated you will be told to go and engage in sex to see if you will get HIV infection or not... (Young policewoman 6, Group 1)*.

Others expressed a fear of seeing a needle with vaccine directed towards the skin as an indication of both physical harm and the possibility of death. One participant pointed with emotion at his upper arm muscle where the vaccine would be injected, believing that it signalled death or survival as the only possible outcomes to expect from the moment the vaccine is injected. He stated:

*When they do it on me pa-a-p [he was pointing at the vaccination site on his upper arm] ... one leg is in [life] and another is out [death] and this makes people worry so much (High ranking policeman 5, Group *5).

Thus, they reasoned that multiple risks could affect their decision about taking part in the HIV vaccine trial. Such worries were fuelled by inadequate knowledge and myths about the vaccine.

#### Time of knowing HIV status

The time of knowing whether one is HIV infected or not was also discussed. The follow-ups and routine HIV-testing associated with participating in the trial will provide early knowledge about whether one is infected, sooner than would be the case if infected through unsafe sex. They viewed the revelation of their status too early as a problem, and therefore people are more afraid of the HIV vaccine than they are of AIDS. One participant remarked:

*Do you know why people are more afraid of the vaccine than AIDS itself? They will become aware earlier [that they are HIV positive]! When she or he comes for testing, she or he will know that he or she is infected. All these will be because of vaccine. Second, is the belief that once I go to get the vaccine I will be counted among the infected. So, fear is fixed in the heart that, what if I die? I have the virus introduced in the body. ... It is better you look for it [HIV through unsafe sex] yourself rather than being implanted (Low ranking policewoman 11, Group 3)*.

The caregiver group had more positive views towards the vaccine trial than the other groups. Their experience had convinced them that it is not possible to be infected with HIV through this vaccine. They did, however, express concern about coping with results from any health test, which is discussed in the following section.

#### Medical check ups: fear versus opportunities

The participants expressed another fear arising from the thorough medical check ups that were a condition for inclusion in the trial. These check ups might reveal that they were infected with HIV or had other life-threatening diseases. A participant expressed:

*You [the researchers] are saying one should undergo testing of everything in the body to see what problems are there. One could have life-threatening condition. Now when he or she is told by another person that he has an untreatable disease, that person will start weakening slowly. Some people can go for testing but cannot take results. I think testing is an issue! (Caregiver 6, Group 8)*.

Others, on the other hand, saw this complete medical check up as an opportunity for receiving free medical services for the diseases that may be discovered:

*Surely, I will be motivated as I think the big issue is to be checked. It's useful for me since if I am found with problems, I will be attended by experts. I will be thankful since I will be given immunity [treatment] (High ranking policeman 7, Group 5)*.

This issue of free medical care as a benefit was discussed in other groups:

*This check up can reveal some diseases that will be treated at the researchers' cost. This is a big advantage, to be treated free and know your status [health status] (Young policeman 2, Group 4)*.

Another participant insisted:

*I think the big issue here is that motivation of health services. The big motivation is to get free medical services for the participant ... (Low ranking policeman 3, Group 6)*.

#### Researchers' assurance and mistrust

Participants expressed trust over what they regarded as the explanations by the skilled researchers about the benefits of vaccine trial and therefore they positioned themselves as recipients. One caregiver said:

*I think the one who can influence is you [the moderator or researchers], with the knowledge you have or the one who gave you education. It is a person with this knowledge who can influence you (Caregiver 2, Group 8)*.

Participants reasoned that the police force may have been selected for the vaccine trial because of the large number of police officers lost through AIDS. Some participants, however, expressed the suspicion that the researchers intended to implant viruses in them instead of preventing the infection. They speculated that the researchers were hiding the truth about the effects of the vaccine:

*They [researchers] are liars. They will plant virus on us! ...Why don't they try with animals? (Low ranking policewoman 12, Group 3)*.

This mistrust seemed to arise from the fact that the vaccine was imported to the country. The participants reasoned that if such a vaccine had been proved safe after testing in animals and then used on human beings elsewhere, there was no need to test it again on Tanzanian police officers. They argued that the researchers in the vaccine project might benefit financially from the trial:

*They [the researchers] already got money from there [donors]. Now to prove they have done the job they are moving around here... A neighbour told me not to engage in the trial (Low ranking policeman 1, Group 6)*.

In summary, participants expressed doubts and fear concerning taking part largely because of inadequate understanding of the Phase I or II HIV vaccine trial. Nevertheless, they saw that the trial offered certain opportunities.

## Discussion

We describe here the reasons that participants are interested in taking part in an HIV vaccine trial. Their interest is tempered by perceived threats that they fear may interfere with their normal lives. They express concern over the possible loss of close relationships and interference with pregnancy norms. They are concerned with the risk of acquiring HIV infection through the vaccine, the time of knowing whether they are infected or not through routine testing, and the implications of undergoing complete medical check ups. The researchers conducting the trial are seen by some as trustworthy and by others as suspicious.

The concern expressed over the possible loss of close relationships is associated with the roles of significant others such as parents, sexual partners, relatives, friends and colleagues. Young participants felt threatened by negative opinions from their significant others more often than older participants, implying that these bonds are crucial in young people's lives. Since this is the age group that has been targeted for Phase I and II HIV vaccine trials, the views and influence of significant others pose an important challenge. Those carrying out trials need to be aware of these concerns, and they need to be aware of the presence of significant others and their influence in a decision whether to participate in the vaccine trial. Previous studies have shown that significant others form an important incentive during participation in an HIV vaccine trial [22,29]. Significant others may exert a powerful influence on a decision to engage in a scientific investigation. Significant others are mentioned as both motivators for, and barriers to, trial participation in a study of community members in South Africa [5]. Thus, significant others can contribute to success or failure in recruiting participants in an HIV vaccine trial. It may be important to involve those who form 'close relationships with the targeted volunteers' from the onset when designing HIV vaccine trials, and encouraging them to share their concerns directly with the trial team.

Pregnancy restriction is mentioned often, suggesting that recruitment of volunteers in the reproductive age group will be a challenge, because of the fear of interference with their reproductive capacities and pregnancy norms. One of the criteria for inclusion in the HIV vaccine trial is an age limit that focuses on young people who expect to have children in the future. Both men and women often voice issues relating to pregnancy restriction. This may be due to the experience of child nurturing, and it may be due to the values placed on children in Tanzania and African contexts [30,31]. Although the pregnancy restriction during the trial is temporary, the participants expressed fear that the vaccine would irreversibly affect their childbearing ability. These concerns have been expressed in other populations [13,14], showing that the question of fertility is a concern in HIV vaccine trial participation in different contexts. More knowledge must be provided to reassure the participants about the trial safety.

The reasoning that participation in an HIV vaccine trial involves implantation of viruses in one's body fuels fear, and participants expressed a worry about getting an HIV infection from a deliberate decision. This fear, however, arises from inadequate understanding and limited information about the contents of the vaccine and how it works. It is generally believed that giving public health information changes knowledge, but we did not assess the extent of knowledge change before and after the workshop to find out whether the information provided had been correctly received. Lack of information about HIV vaccines and confusion about how vaccines work has been noted in another study [32]. Similarly, a study in Uganda reported fears about deliberate infection with HIV from participation in a trial [12]. Other studies have also reported a fear of vaccine-induced HIV infection [4,6,14,16,33] and a fear of detrimental effects on participants' lives [34,35].

The discovery of one's HIV status as a consequence of the routine testing in the trial aroused fear. However, approaches to HIV prevention in Tanzania have suggested that knowledge of one's HIV status can empower individuals to take precautions to protect against either acquiring or transmitting the disease [36]. Similarly, if many people prefer to remain ignorant of their HIV status as long as possible following accidental exposure to infection, this will undermine government efforts to prevent new infections. It is important to stress the importance of early HIV testing and to promote safe sex during the recruitment of volunteers to future HIV vaccine trials.

Complete medical check ups that follow from participation in the trial provide the opportunity of free healthcare, but this is tempered by the fear of discovering that one is suffering from an unknown disease. The threat of finding out that one is suffering from a life-threatening disease may give rise to a feeling of panic, a feeling that one is facing death too early. The worry experienced by participants is exacerbated by the lack of adequate insurance in Tanzania and the expected payout from the insurance scheme. Similarly, compensation for families in the event of a poor trial outcome is considered important in south India [37]. Among parents in Gambia, a decision to let their children participate in a trial involved a sense of balance between the benefit of free medical treatment and the danger of one's child being drained of blood [17]. Getting free healthcare in a trial appears to be important for the willingness of adolescents to take part [38]. Participants must weigh the benefits against the risks of participating in a trial. It is, therefore, important that the consequences of taking part in a trial are transparent.

Mistrust of the researchers adds challenges to scientific investigations. Doubt about the integrity of researchers can seriously interfere with recruitment, not only to an HIV vaccine trial but also to future trials with other products that are not related to an HIV vaccine. Other studies have revealed mistrust of scientists conducting trials [8,14,39-41]. Trust for researchers is related to the question of how donor funds are used, and this is not surprising. AIDS-related funding for low-income countries such as Tanzania is provided by the international community (donors) [42], and usually reaches the projects that spend it via one (or more) of four main funding streams: donations from national governments; multilateral funding organisations; the private sector; and domestic spending. Mismanagement may not be so much a case of corruption, but rather that money is simply not being spent wisely, or used to its maximum effect. A government department, for example, may spend excessive amounts on paying for experts to travel to projects and stay in expensive accommodation, when the money could be better spent carrying out work on the ground. Such phenomena may lead people to believe that the researchers are benefiting from AIDS-related funding. Hence, the concerns raised by our participants (which may be shared by the whole nation, not only the participants) are legitimate and need attention. Conversely, participation in the trial is viewed as an important step in helping researchers to find an effective HIV vaccine [34].

We cannot generalize the results presented here to other populations, but the qualitative approach we have used has enabled us to determine the reasons behind the participants' decision whether to take part in an HIV vaccine trial. Focus group discussions (FGDs) were useful in helping participants to explore and clarify their views in ways that would be less accessible in a one-to-one interview. Participants were able to explore issues that were important to them in their own vocabulary as shown in the quotes. (It is possible that one-to-one interviews are useful in providing an in-depth description of some statements). The use of FGDs shaped the data by generating a shared perspective. Group interaction encouraged participants to mention sensitive issues and issues that researchers had not thought about. The use of FGDs obtains data more rapidly than one-to-one interviews and it is thus cheaper [43]. In addition, the use of an approach using interpretive descriptions (ID) can guide study design. It assisted in generating practice-relevant data and examining the responses of the participants with the goal of identifying themes and patterns, and accounting for variations between groups. ID is a relatively new qualitative method in health care [44], that requires the user to interpret their findings carefully, in order to retain the original meaning of the participants' perspectives.

## Conclusions

The opinions and reasons for deciding whether to take part in an HIV vaccine trial among police officers are important. These findings have immediate implications for how Phase I and II HIV vaccine trials among police officers in Dar es Salaam, Tanzania are designed. The use of the qualitative research method described here enabled us to elicit the perceptions and understanding of HIV vaccine trials in a local context from potential volunteers. The results can be used when modifying the trial recruitment materials to ensure that potential volunteers and the general population are given correct information before Phase I and II HIV vaccine trials are implemented. In particular, the role of significant others and a mistrust of researchers and vaccine products are important issues to address when designing future trials. These issues should be addressed from the onset of trial design and monitored throughout the trials because they may also influence whether those who volunteer for trials complete the trial. Fear of vaccine side effects requires that potential volunteers be given extensive knowledge and reassurance, to ensure that they adequately understand the trial and to increase the probability that those who volunteer complete the trial. Further studies are planned that will follow up these results and examine their significance for policy formulation when conducting trials with different groups in similar settings.

## Competing interests

The authors declare that they have no competing interests.

## Authors' contributions

EAMT conceived the study, coordinated data collection, carried out analysis and drafted the manuscript. AK participated in the study design, data analysis and critically reviewed the final draft. AT, TWK, MB and ES were involved in study design and critically reviewed the manuscript. JM participated in the study design, data collection and reviewed the manuscript. All authors read and approved the final manuscript.

## Pre-publication history

The pre-publication history for this paper can be accessed here:

http://www.biomedcentral.com/1471-2458/10/292/prepub
